# Catalpol Mitigates Alzheimer's Disease Progression by Promoting the Expression of Neural Stem Cell Exosomes Released miR-138-5p

**DOI:** 10.1007/s12640-022-00626-z

**Published:** 2023-01-03

**Authors:** Shengxi Meng, Huize Chen, Chunjun Deng, Zeyu Meng

**Affiliations:** 1 Department of Traditional Chinese Medicine, Shanghai Sixth People’s Hospital Affiliated to Shanghai Jiao Tong University School of Medicine, Xuhui District, No.600 Yi Shan Road, Shanghai, 200233 China; 2 Second Clinical Medicine College, Heilongjiang University of Chinese Medicine, Harbin, 150040 China

**Keywords:** Alzheimer’s disease, Catalpol, Neural stem cell, Exosomes, miR-138-5p

## Abstract

Alzheimer’s disease (Alzheimer’s disease, AD) is a neurodegenerative disease characterized by senile plaque deposition and neurofibrillary tangles. The pathogenesis of AD is complicated and the drugs used to treat AD are single-targeted drugs, which can only improve or alleviate the symptoms of patients, but cannot delay or prevent the progress of the disease. Because of its ability to act on multiple targets, multiple systems, multiple links, and multiple pathways, Chinese herbal compound prescriptions have shown unique advantages in the research and treatment of AD. Our previous study has demonstrated the protect role of the Chinese medicine Rehmannia in AD. However, the underlying mechanism remains unclear. In the present study, both in vitro and vivo experiments were employed, and we found Catalpol (Ca), the main extract of Rehmannia, could mitigate AD progression both in vitro and in vivo by promoting miR-138-5p level in neural stem cell secreted exosomes

**Supplementary Information:**

The online version contains supplementary material available at 10.1007/s12640-022-00626-z.

## Introduction

Alzheimer’s disease (AD) is an irreversible neurodegenerative disease, with insidious onset, gradual development, and progressive aggravation of symptoms, characterized by memory loss and cognitive dysfunction (Rochoy et al. 2019). The pathological features of AD are diffused brain atrophy, characteristic neurofibrillary tangles, senile plaque deposition in brain tissue, and neuron loss (Minati et al. 2009). The pathogenesis of AD involves abnormal accumulation of Aβ, hyperphosphorylation of Tau protein, oxidative stress, inflammation, and immune abnormalities; with the deepening of research, new factors leading to the progression of AD are gradually being discovered (Sanabria-Castro et al. 2017), which indicated the complexity of AD pathogenesis leads to the lack of specific drugs in AD treatment. The drugs currently used to treat AD include cholinesterase inhibitors, NMDA receptor antagonists, antioxidant, neurotrophic, and immunotherapy drugs (Tiwari et al. 2019). However, most of drugs, such as tacrine and donepezil hydrochloride, are single-targeted drugs, which can only improve or alleviate the symptoms of AD patients to a certain extent, but cannot alleviate or prevent the progress of the disease (Li et al. 2018; Ismaili et al. 2017); thus, multi-targeted and more efficient drugs are urgently needed. Traditional Chinese medicine compounds have shown unique advantages in the treatment of AD due to their effects on multiple targets and multiple pathways (Klimova and Kuca 2017; Shi et al. 2017), such as Rehmannia, which has been shown promising efficacy in AD treatment; however, the mechanism of Rehmannia remains unknown.

In clinical research, stem cell–based approaches, including the use of neural stem cells (NSCs), have shown potential for the regenerative treatment of various diseases including AD (Hayashi et al. 2020; Zhao et al. 2018). Stem cell–based therapy is based mainly on cell replacement and the induction of paracrine effects to replace damaged cells, reduce cell death, and provide trophic support for host cells (Fan et al. 2020; Harrell et al. 2019; Marei et al. 2018). However, preclinical studies have suggested that very few (< 1% of injection) cells could survive over 4 weeks after transplantation, because of the hostile, injured microenvironment. Furthermore, the association between the therapeutic benefits of transplantation and the paracrine effects of grafted cells is not yet known (Kwak et al. 2018; Wang et al. 2018). In recent years, studies have confirmed that paracrine signals are related to extracellular vesicles (EVs), which have been implicated as mediators of paracrine benefits and are involved in cell communication (Rad et al. 2019; Webb et al. 2018). EVs, produced by all living cells, include microvesicles (50–1000 nm) and exosomes (40–200 nm). Exosomes are released into extracellular fluids by living cells; contain proteins, lipids, and genetic materials (mRNA, ncRNA, etc.), and play essential roles in intercellular communication by transferring exosomal protein and RNA cargos between the source and target cells (Reiner et al. 2017; Carnino et al. 2019). Emerging data have shown that exosomes have been successfully tested in preclinical models of stroke, myocardial infarction/reperfusion injury, and hind limb ischemia (Harane et al. 2018; Zarà et al. 2020). Furthermore, anti-tumor therapies based on EVs derived from dendritic cells have entered phase II human clinical trials (Fernández-Delgado et al. 2020).

MiRNAs are highly conserved, noncoding small RNAs and about 22 nucleotides in length. The role of miRNAs is mainly achieved through combination with the base of the 3′-untranslated region (3′-UTR) of the target gene mRNAs to suppress their expressions by degradation and translational inhibition (Chen et al. 2015). A single miRNA can regulate hundreds of target gene mRNAs, and a single target gene mRNA can be regulated by several different miRNAs (Jackson and Standart 2007), such as microRNA-138-5p (miR-138-5p); it suppresses MnCl_2_‐induced autophagy by targeting SIRT1 in SH‐SY5Y cells (Ma et al. 2019a), reverses gefitinib resistance in non-small-cell lung cancer cells via targeting G protein–coupled receptor (Gao et al. 2014), and inhibits proliferation and motility of breast cancer by targeting PD-L-1 (Rasoolnezhad et al. 2021). In recent years, miRNA-138-5p was found highly expressed in nervous system and play an important role in regulating memory and axon regeneration (Bao and Cao 2019). Dysregulation of miR-138-5p promotes the progression of intervertebral disc degeneration and inhibits axon regeneration (Wang et al. 2016). A recent study also shows that miR-138-5p controls the shape and size of dendritic spines in rat hippocampal neurons during development and thereby influences long-term memory (Maza et al. 2020). Following injury, miR-138-5p participates in axon regeneration in peripheral nerve and promotes neuroplasticity through the regulation of vimentin in the damaged spinal cord (Sullivan et al. 2018). However, whether miR-138-5p involved in the progression of AD remains unknown.

Our recent study demonstrated that the traditional Chinese medicine Rehmannia is effective in the treatment of Alzheimer’s disease (Meng et al. 2020). This study aims to explore the underlying mechanisms of Rehmannia function in vitro and vivo experiments. We confirmed that miR-138-5p was selectively packed into exosomes and interfered with neurons apoptosis and AD progression by targeting the 3′UTR of Tau. Furthermore, we demonstrated that Ca, the extract of Rehmannia, could promote miR-138-5p level in NSCs secreted EVs, and thus exert its biological function in AD treatment.

## Material and Methods

### Animals

PSAPP-Tg (*APPSwePS1d9*, Jackson Laboratory, number 34832) and WT C57BL/6 mice (used as control) were purchased from Jackson Laboratory (Bar Harbor, ME, USA) and bred in-house. PSAPP-Tg are double transgenic mice expressing a chimeric mouse/human amyloid precursor protein (Mo/HuAPP695swe) and a mutant human presenilin 1 (PS1-dE9), both directed to CNS neurons. Both mutations are associated with early-onset Alzheimer’s disease, and regularly used as in vivo models (Jankowsky et al. 2004). Mice were reared (3–5/cage) with free access to food and water. PSAPP (*APPSwePS1d9*) mice were confirmed by genomic DNA extraction and PCR. Catalpol (Ca), purchased from Nanjing Jingzhu Biotech Ltd. Co (Nanjing, China), was given by intragastric administration daily for 4 weeks (120 mg/kg) beginning from the 9 week of PSAPP-Tg mouse. All animal protocols were approved by the Shanghai Jiao Tong University Affiliated Sixth People’s Hospital.

### Establishment of the In Vitro AD Model

Cultured human neuroblastoma SH-SY5Y cells were purchased from the Chinese Academy of Sciences. When the SH-SY5Y cells grew to 80%, the experiments were started. SH-SY5Y cells were resuspended to the appropriate concentration, and amyloid β protein fragment 1–40 (Aβ1–40) (#A1075, Sigma-Aldrich, St. Louis, MO, USA) was added to the fresh complete culture medium up to a final concentration of 5 μmol/L. Cells were incubated in the cell culture incubator with 5% CO_2_ at 37 °C for 24 or 48 h.

### Mouse NSC Isolation and Enrichment

Mouse cortical NSCs were isolated from mouse fetal brain tissue as previously described (Ma et al. 2019b). Briefly, cortical tissues were isolated from embryonic day 13.5 (E13.5) mice and triturated physically 15–20 times. Dissociated tissues were filtered through a 40-μm filter, and single cells were cultured in substrate-free tissue culture flasks for the formation of neurospheres in NSC proliferation medium, containing NeuroCult® NSC Basal Medium (Stem Cell Technologies), NeuroCult® NSC Proliferation Supplements (Stem Cell Technologies), 20 ng/mL FGF2 (BioWalkersville), 20 ng/mL EGF (BioWalkersville) and 2 μg/mL heparin (Sigma), 1% N2 supplement (Gibco), 2 mM l-glutamine, and 100 U/ml penicillin and streptomycin. Primary neurospheres were collected, centrifuged at low speed to remove flowing cells in the supernatant, dissociated into single cells with Accutase (Sigma) for 5 min, and re-plated for a second round of neurosphere formation. Enriched NSCs were harvested after three rounds of neurosphere formation. These cells were continue cultured in NSC proliferation medium, with 5% CO_2_ at 37 °C, the medium was changed every 3 days.

### NSC Differentiation and Characterization

The differentiation of NSCs was as previously described (Ma et al. 2019b). Briefly, to induce NSCs into neuron, astrocyte, and oligodendrocyte, 5 × 10^4^ NSC proliferation medium–cultured NSCs were transferred into neuron differentiation medium, astrocyte differentiation medium and oligodendrocyte differentiation medium, respectively, and replace medium every 2 days, continue culturing for 2 weeks. Neuron differentiation medium (50 ml): 1% B27 serum free supplement (Life Technologies), 1% N2 supplement (Gibco), 5 μg/ml insulin, 20 ng/ml BDNF (Gibco), 20 ng/ml CNTF (Gibco), 10 μm Forskolin (Sigma), 25 mM l-glutamic acid, 200 mM l-glutamine, 500 μl 100 × Pen/Step, 50 ml Neurobasal medium (Life Technologies). Astrocyte differentiation medium (50 ml): 1% N2 supplement, 2 mM GlutaMAX (Gibco), 1% FBS, 50 ml DMEM. Oligodendrocyte differentiation medium (50 ml): 10 ng/ml FGF-basic (R&D systems), 10 ng/ml PDGF (Peprotech), 10 nM Forskolin, 50 ml DMEM/F12 (Gibco). The characterization of NSCs and its ability to differentiate into neurons, astrocytes, and oligodendrocytes were confirmed using cyto-immunofluorescence. Cells were fixed with PBS (0.1 M) containing 4% paraformaldehyde at room temperature (RT) for 15 min and permeabilized with 0.1% Triton X-100 in PBS for 5 min. Briefly, cells were preincubated for 10 min with 3% normal goat serum and 2% bovine serum albumin in PBS containing 0.4% Triton X-100 to block background immunostaining. For immunofluorescence staining, NSCs or differentiated cultures were incubated overnight with the corresponding antibodies: Nestin (ab105389, Abcam), Sox2 (ab92494, Abcam), Musashi1 (ab52865, Abcam) as markers for NSCs; Tuj1 (ab18207, Abcam) and Map2 (ab5392, Abcam) for neurons, peripherin (ab4666, Abcam), and GABA (ab216465, Abcam) for astrocytes; CNP (ab6319, Abcam) and MBP (ab254026, Abcam) for oligodendrocytes. For visualization, the primary antibody was developed by incubating with Alexa Fluor 488– or 594–conjugated secondary antibodies for 1 h at RT against the corresponding species. The cells were analyzed with a laser scanning confocal microscope equipped with Fluoview SV1000 imaging software (Olympus FV1000), or with an Olympus BX51 microscope.

### Preparation and Characterization of Exosomes

Exosomes were isolated from NSCs as previously described (Zhong et al. 2020). The isolation method comprised an additional centrifugation step to remove small cell debris followed by ultracentrifugation at 100,000 × *g* for 1 h to generate an exosome pellet. Afterwards, the pelleted exosomes were resuspended in PBS. The concentration and size distribution of exosomes were confirmed by nanoparticle tracking analysis (NTA) using NanoSight NS300. The morphology was observed by transmission electron microscopy. To detect exosome markers and negative markers, Western blotting was performed with anti-CD63, anti-HSP70, anti-TSG101, and anti-tubulin antibodies.

### NSCs or Exosome Treatment

In total, 1 × 10^5^ SH-SY5Y cells were seeded into the lower chambers. For the cell treatment, approximately 5 × 10^5^ NSCs were seeded into the upper chambers of 6-well cell culture inserts. Exosomes were added to the culture medium at 2 μg of exosomes per 1 × 10^5^ recipient cells. GW4869 is a neutral sphingomyelinase inhibitor, and is the most widely used pharmacological agent for blocking exosome generation (Reiner et al. 2017). GW4869 inhibits the inward budding of multivesicular bodies and release of mature exosomes; thus, GW4869 was used in the present study to inhibit exosome secretion from NSCs. Twenty micromolar of GW4869 (#D1692, Sigma) was used to treat NSCs.

### Flow Cytometry

To analyze the apoptosis population of SH-SY5Y cells, flow cytometry using annexin V-FITC and propidium iodide (PI) staining was performed using an Annexin V/PI detection kit (#559763, BD Biosciences, San Jose, CA, USA) with a FACSCalibur flow cytometer. Neuronal cells were trypsinized, collected, and washed with PBS. Cells were counted and 1 × 10^6^ cells were suspended in 1 ml cold binding buffer (10 mM HEPES/NaOH, pH 7.4, 140 mM NaCl, and 2.5 mM CaCl2). Cells were aliquoted into 1.5 ml tube at 1 × 10^5^ cells per tube, and were incubated with 5 μl of annexin V-FITC and 2 μg/ml of PI at room temperature for 15 min. After incubation, 400 μl of binding buffer was added and flow cytometric analysis was performed. FITC and PI fluorescence were passed through 520- and 630-nm bandpass filters, respectively, and the data were analyzed using Flowing Software. The apoptotic rate was calculated as a percentage of Q2 + Q4 quadrants. Six replicates were performed for each group.

### Cell Viability Test

The cytotoxicity of Ca was assessed using the methyl thiazolyl tetrazolium (MTT) assay. Viable cells have the ability to reduce the yellow MTT to purple formazan crystals by mitochondrial dehydrogenase enzymes. The SH-SY5Y or NSC cells were harvested and seeded on 12-well culture plates. A MTT stock solution was prepared fresh as 5 mg/ml in PBS and filtered through a 0.22-μm filter. After 48 h of culture with Ca, the MTT solution was mixed with NSC proliferation medium without phenol red in the ratio 1:9, added to each culture well, and incubated in the dark for 3 h at 37 °C. Then, the formazan crystals were dissolved in isopropanol. The absorbance of the resulting purple solution was spectrophotometrically measured at a wavelength of 570 nm (Spectra Academy, K-MAC, Korea). Each experiment was repeated three times. The viability of the corresponding cells was calculated as the percentage of MTT reduction and the absorbance of control cells was assumed as 100%.

### Tissue Immunofluorescence

Hippocampal slices were prepared as previously described (Lin et al. 2018). Mice were anesthetized with isoflurane before decapitation. The brain was quickly removed and immersed for 2 min in ice-cold low-calcium “modified” artificial cerebrospinal fluid (mACSF) composed of 119 mM NaCl, 2.5 mM KCl, 1.3 mM CaCl_2_, 2.7 mM MgSO_4_, 1 mM NaH_2_PO_4_, 26 mM NaHCO_3_, and 10 mM glucose, continuously bubbled with 95% O_2_ plus 5% CO_2_. The dorsal hippocampus was dissected out and cut in ice-cold mACSF with a vibratome (Leica VT1000S; Nussloch) into 5–15-μm-thick slices from the middle part of the hippocampus. Floating sections were blocked with 10% goat serum and incubated with rabbit anti-Cav-1 (1:1000, Cell Signaling, #3267, USA) at 4 ℃ overnight. Slices were then incubated with rabbit-specific fluorescence secondary antibody in the dark for 1 h and counter-stained for 5 min with DAPI (Beyotime Biotechnology, China) and observed by a fluorescence microscope (DTX500; Nikon Corporation, Tokyo, Japan). The fluorescence intensity was quantified and analyzed with the ImageJ 1.5 software (Bethesda, Maryland, USA).

### TUNEL Assay

To detect apoptotic cells in rat brain tissue samples, TUNEL assay using a DeadEnd™ Fluorometric TUNEL system (Promega Corporation, Madison, WI, USA) was performed according to the manufacturer’s protocol. Cell nuclei with red fluorescent staining was defined as apoptotic cells. To quantify TUNEL‑positive cells, the number of red fluorescence‑positive cells was imaged by a fluorescence microscope (DTX500; Nikon Corporation, Tokyo, Japan) and counted in five random fields.

### miRNA Mimics/Inhibitors and Transfection

The mimics control, MiR-138-5p mimics, inhibitor control, and miR-138-5p inhibitor were purchased from GenePharma (GenePharma Co., Ltd., Shanghai). Transfection of miRNA mimics/inhibitors was performed using the Lipofectamine 2000 reagent (Invitrogen) according to the manufacturer’s instruction.

### miR-138-5p Knockdown In Vivo

The procedure was carried out as previously described (Barbash et al. 2013). Briefly, the mice were anesthetized and fixed to a stereotaxic apparatus. The left lateral ventricle of the brain was stereotaxically implanted with a brain infusion cannula (bregma: − 0.22 mm; dorsoventral: 3 mm; lateral: 1 mm) connected with a microosmotic pump (Model 1004, Alzet, Cupertino, CA, USA). Continuous intracerebroventricular infusion of miR-138-5p inhibitor and negative control of scramble RNA for miR-138-5p inhibitor (Dharmacon and Shanghai GenePharma Co, China) was delivered to the brain at a rate of 0.2 ml/minute.

### Western Blot

Western blot was carried out for exosome and cell lysates as previously described (Wei et al. 2020). Briefly, exosomes or cells were lysed in RIPA lysis and extraction buffer (Thermo Scientific). Protein concentration was determined using the BCA (bicinchoninic acid) Protein Assay Kit (Pierce). Blots were incubated with primary antibodies for Caspase3 (1:3000, ab32351, Abcam), Bcl-2 (1:1000, ab32124, Abcam), Bax (1:1000, ab32503, Abcam), CD63 (1:1000, ab134045, Abcam), Hsp70 (1:1000, ab2787, Abcam), TSG101 (1:1000, ab125011, Abcam), Tau (1:1000, ab254256, Abcam), and Tubulin (1:3000, ab7291, Abcam) overnight at 4 °C. Corresponding HRP-conjugated anti-rabbit or anti-mouse (1:10,000, Pierce) secondary antibodies were incubated for 1 h at RT. Bands were visualized with an ECL kit (Pierce). The density of the immunoblots was determined by image lab software and analyzed using Image J program.

### Luciferase Reporter Assay

Tau fragment with the predicted binding site to miR-138-5p binding site was cloned into a psiCHECK-2 luciferase reporter to form the reporter vector psiCHECK-2-Tau-wild-type (Tau-WT). The PCAT-1- miR-138-5p binding site was mutated as indicated and named as psiCHECK-2-mutated-type (Tau-MT). Transfection of psiCHECK-2- Tau-wt or psiCHECK-2-Tau-mut was done along cotransfected with miR-138-5p mimics or control with Lipofectamine 2000. A 96-well plate was used to seed 5000 293 T cells per well. Following 48-h transfection, Dual-Luciferase Reporter Assay System (Promega) was applied by adherence to the prescribed procedures. Triplicate assays were conducted.

### RNA Immunoprecipitation Assay

The RNA immunoprecipitation (RIP) assay was performed using 293 T cells and the Magna RIP™ RNA-Binding Protein Immunoprecipitation Kit (Millipore), according to the manufacturer’s instructions. Briefly, the cultured 293 T cells were lysed using RIPA buffer and subsequently incubated with RIP buffer, and then RNAs magnetic beads (Invitrogen) were conjugated to anti-Ago2 antibody (Millipore, Bedford, MA) or the negative control anti-IgG antibody (Millipore). Following the total RNA extraction, the precipitated complex was subjected to qRT-PCR.

### Quantitative Polymerase Chain Reaction

The mRNA and miRNA were isolated from cell and tissue samples using miRCURY RNA isolation kit (Exiqon, Woburn, MA). cDNA was synthesized using miScript II RT kit (Qiagen, Valencia, CA). Transcripts were amplified using gene-specific primers and SYBR green PCR kit (Qiagen, Valencia, CA) with the ABI7500 (Applied Biosystems, Waltham, MA). All RT-qPCR results measured in each sample in triplicate and no-template blanks were used for negative controls. The primers for miR-138-5p were 5′-AGCTGGGTTGTGAATCAGGCCG-3′ (sense) and 5′-TGGTGTCGTGGAGTCG-3′ (antisense); The Tau primers were 5′-CCAGTCCAAGTGTGGCTCAAAG-3′ (sense) and 5′-GCCTAATGAGCCACACTTGGAG-3′ (antisense). The GAPDH primer forward primer were 5′-ACTCCACTCACGGCAAATTC-3′ and the reverse primer is 5′-TCTCCATGGTGGTGAAGACA-3′; the PCR primers for U6 were 5′-GCTTCGGCAGCACATATACTAAAAT-3′ (forward) and 5′-CGCTTCACGAATTTGCGTGTCAT-3′ (reverse). Amplification curves and gene expressions were normalized to the house-keeping gene GAPDH (for mRNA) and U6 snRNA (for miRNA). The thermal cycling profile featured a pre-incubation step of 94 ℃ for 10 min, followed by 40 cycles of denaturation (94 ℃, 15 s), annealing (55 ℃, 30 s), and elongation (72 ℃, 20 s). Melting curves were subsequently generated (94 ℃ for 15 s, 50 ℃ for 30 s, slow heating to 94 ℃ in increments of 0.5 ℃). Melting-curve analyses confirmed that only single products had been amplified. The thresholds calculated using the software were used to calculate specific mRNA expression levels using the cycle-at-threshold (Ct) method, and all results are expressed as fold changes (compared to control) for each transcript, employing the 2^−ΔΔCt^ approach.

### Rotarod Test

Subjects were tested for motor coordination and learning on an accelerating rotarod (Ugo Basile, Stoelting Co.). On a first test session, mice were given three trials, with 45 s between each trial. An additional trial was given 48 h later. The initial speed was set at 3 rpm, with a progressive increase to a maximum of 30 rpm across a total 5-min trial. Measures were taken for latency to fall from the top of the rotating barrel.

### Statistical Analyses

All results are the means of at least three independent experiments ± SD. Data from two groups were evaluated statistically by two-tailed, paired, or unpaired student *t* test. Significance was considered when *P*-value < 0.05.

## Results

### In Vitro AD Model Construction

**Fig. 1:**
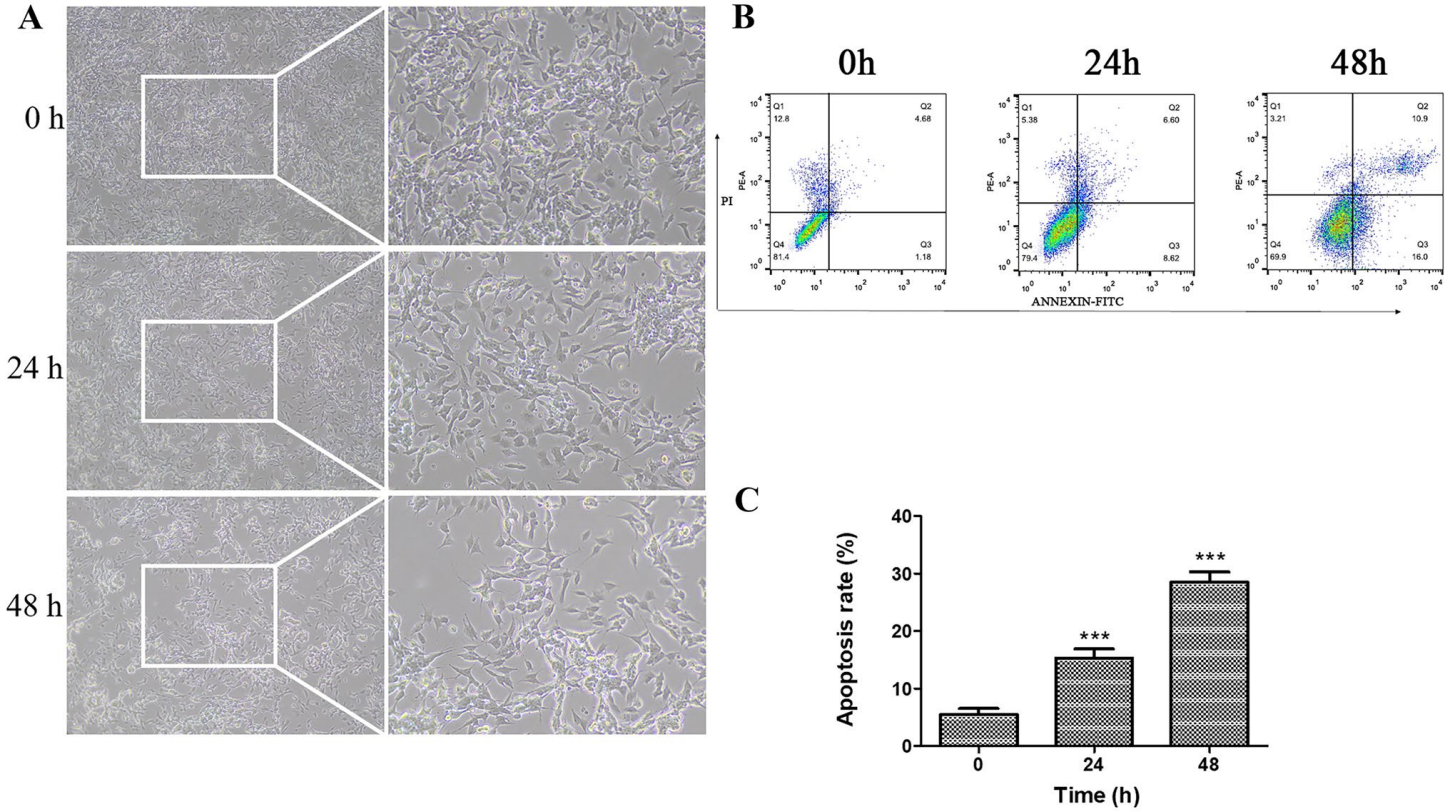
AD model establishment with Aβ1–40. Human neuroblastoma SH-SY5Y cells were supplemented with 5 μmol/L amyloid β protein fragment 1–40 (Aβ1–40) for 48 h, before (0 h), 24 h or 48 h after Aβ1–40 supplementation, cell phenotype were observed (**A**). At the three timepoint, the apoptosis of these cells was detected with flow cytometry (**B**). Quantification of **B** showed in **C**. ***p < 0.001 vs 0 h

The in vitro AD model was established with Aβ1–40 as previously reported (Liu et al. 2018). The first approach to validate our model was to evaluate the morphology of the cells. Normally, SH-SY5Y cells are large and bright and round or oval in shape. After exposure to Aβ1–40, the cell number was reduced, the morphological appearance was changed correspondingly, and cell body shrinkage, rough cell surface, and retracted cell neurites were observed. As the exposure time increased, the morphological changes became more obvious (Fig. 1A). The flow cytometry results showed a significant, time-dependent increase in cell apoptosis in the AD model (Fig. 1B, C), which indicated that the in vitro AD model with SH-SY5Y cells was successfully established by Aβ1–40.

### NSCs and NSCexo Protect AD Model Cell from Apoptosis

**Fig. 2:**
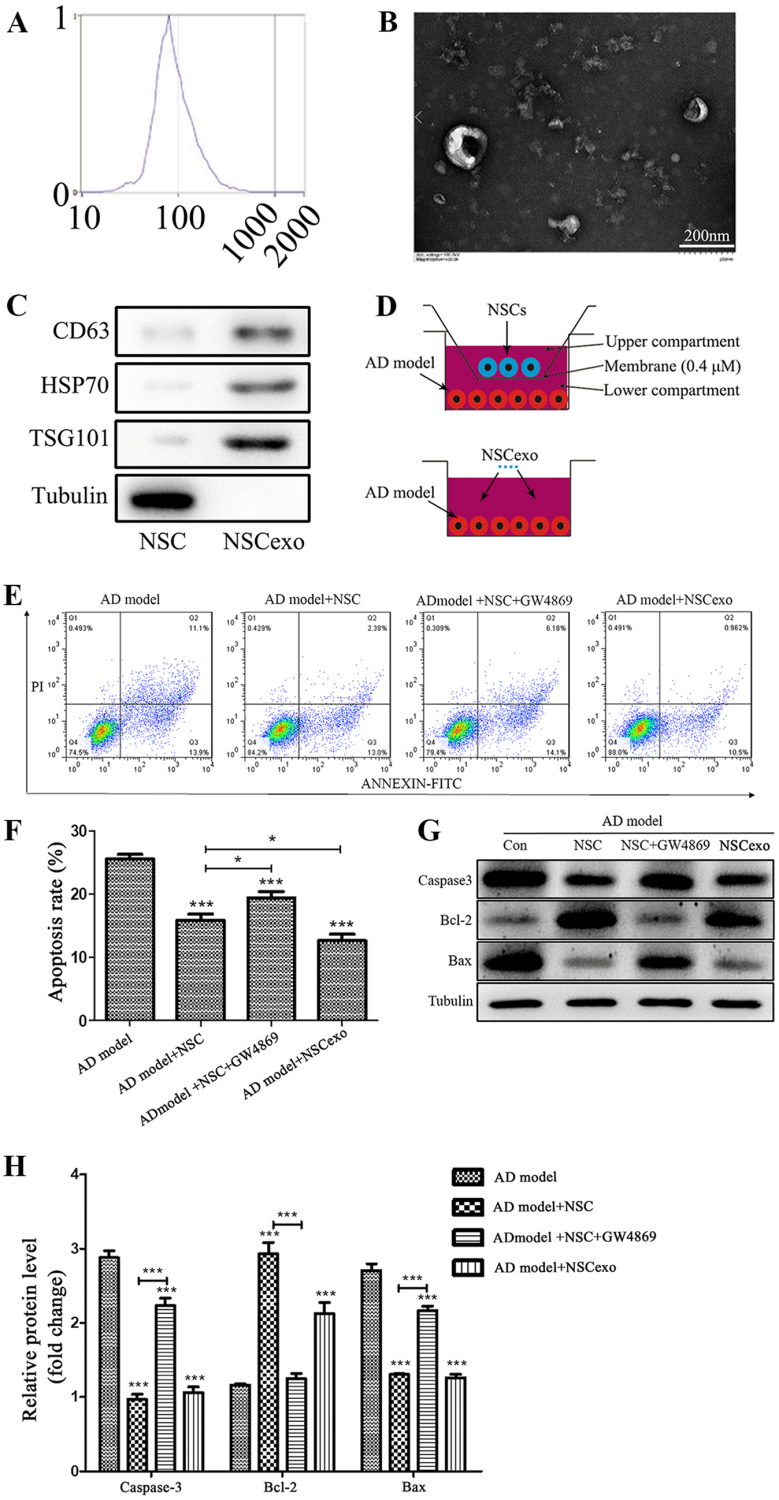
Characterization of NSC-derived small extracellular vesicles and the function of NSCexo. **A** Particle size distribution measured by dynamic light scattering. **B** Small extracellular vesicle morphology revealed by transmission electron microscopy (TEM), scale bars: 200 nm. **C** Western blot analysis of specific small extracellular vesicle surface markers including CD64, HSP70, and TSG101, with the Tubulin as negative control. **D** Representative of the Transwell non-contact coculture assay system that used with NSCs and the in vitro AD model, and the contact coculture assay system that used with NSCexo and the in vitro AD model. **E** Flow Cytometry was used to detect the apoptosis of SH-SY5Y cells that supplemented with 5 μmol/L Aβ1–40 for 48 h (AD model), AD model cocultured with NSCs (AD model + NSC), together with the exosome inhibitor GW4869 (AD model + NSC + GW4869), or AD model with NSCexo. quantification of **E** showed in **F**. **G** Western-blot was employed to detect the accumulation of apoptotic related markers, including Caspase3, Bcl-2, and Bax in these cells, Tubulin was used as internal control. Quantification of **G** showed in **H**. *p < 0.05, ***p < 0.001 vs AD model or the indicated group

To analyze the impact of NSC-derived exosomes (NSCexo) on in vitro AD model, we first characterized NSCs and their derived exosomes. The NSCs were successfully isolated and were identified by molecular markers, morphological observation, and differentiation ability. The cell morphology of NSCs was observed under an inverted microscope with neurosphere morphology (sFig. 1A). NSCs positively expressed the cell surface markers Nestin, SOX2, and Musashi1 (sFig. 1B-D). Then, the multilineage differentiation capability of NSCs was analyzed based on their ability to differentiate into neurons, astrocytes, and oligodendrocytes (sFig. 1E-G). Subsequently, NSCexo were analyzed for size distribution and numbers with a NanoSight system. NTA indicated a homogenous population with low dispersity and with a peak in particle size at ~ 90 nm (Fig. 2A). In addition, a bilayer cup-shaped morphology was detected under electron microscope (Fig. 2B). Western blot analysis showed the expression of the exosomal marker proteins CD63, Hsp70, and TSG101, while the expression of tubulin was negative (Fig. 2C). Therefore, the above results demonstrated the efficacy of the extraction protocol.

Once secreted, exosomes are internalized by neighboring or distant cells. To determine the effect of exosomes, we employed a Transwell coculture system for NSCs and the in vitro AD model in which the cells were separated by a porous membrane with 0.4-μM pores. Additionally, a contact coculture system for exosomes and the in vitro AD model was established (Fig. 2D). The AD model cells were treated with NSCs or NSCexo. As expected, the apoptosis rate of the AD model cells cocultured with NSCs and NSCexo was significantly reduced (Fig. 2E, F). In addition, we assessed the accumulation of caspase3, Bcl-2, and Bax, as shown in Fig. 2G and H. these apoptosis-related markers were significantly reduced in NSCs and NSCexo co-cultivated SH-SY5Y cells. However, these protective effects were inhibited after pretreatment with the exosome inhibitor GW4869. These findings indicated that NSCexo protected the in vitro AD model against the damaging effect. In summary, NSCs and NSCexo showed similar neuroprotective effects.

### miR-138-5p Expression in the AD Model Cells Were Upregulated by NSCexo

**Fig. 3:**
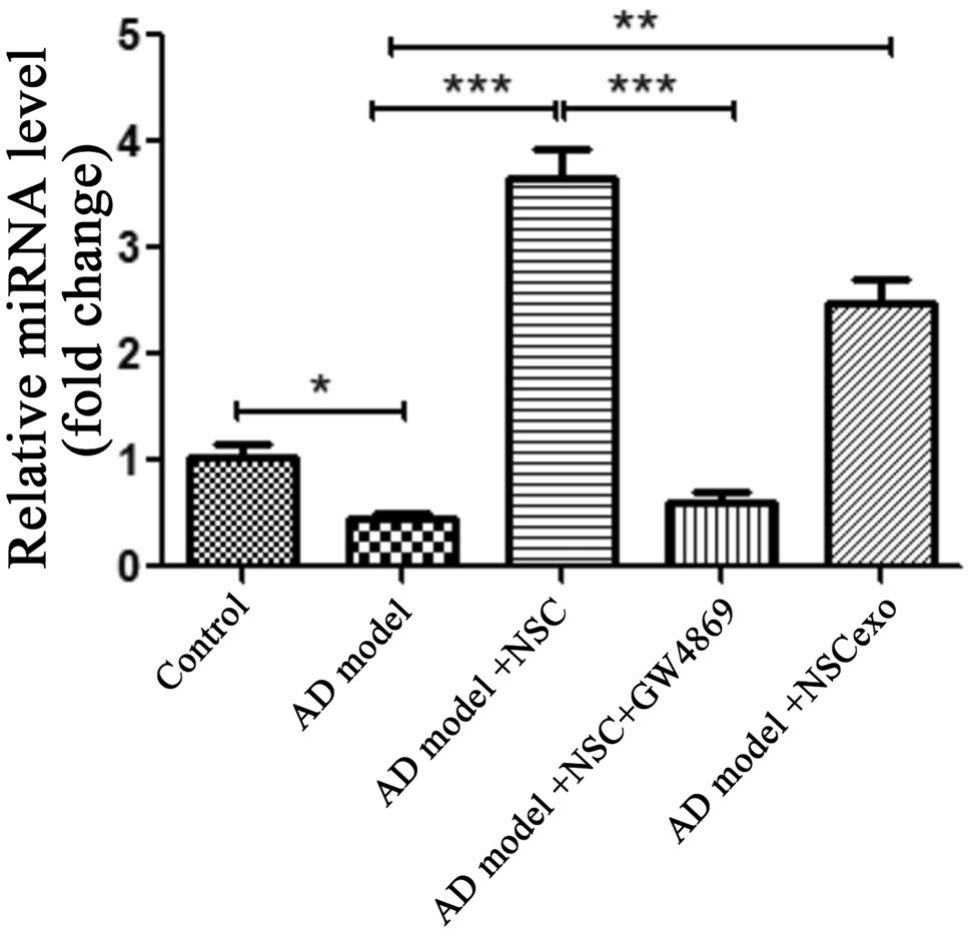
miR-138-5p level detection in AD model cells. RT-PCR was performed to detect miR-138-5p levels in SH-SY5Y cells that supplemented with 5 μmol/L Aβ1–40 for 48 h (AD model), AD model cocultured with NSCs (AD model + NSC), together without the exosome inhibitor GW4869 (AD model + NSC + GW4869), or AD model with NSCexo. *p < 0.05, **p < 0.01, ***p < 0.001 vs AD model or the indicated group

The exosome-encapsulating miRNA is regarded as a novel and important part of the intracellular communication mechanism, and various studies have shown that miR-138-5p plays a role in different cellular processes, ranging from cancer progression and neuron homeostasis (Wang et al. 2016; Zhao et al. 2016). Given its role in basic cellular functions found in our previous study (Wang et al. 2016), it is not surprising that miR-138-5p has the potential to inhibit cell apoptosis. To determine the impact of miR-138-5p on AD, we first detected the expression of miR-138-5p of the in vitro AD model, and found a significant decrease in miR-138-5p (Fig. 3A). Then, the in vitro AD model was co-cultured with NSCs, and we found the increased miR-138-5p in the AD model cells. However, when NSCs in the co-cultured system were pretreated with GW4869, the miR-138-5p level in the AD model cells was significantly reduced. Meantime, it was noted that NSCexo alone could effectively promotes miR-138-5p level in the in vitro AD model. These data indicate that the increased level of miR-138-5p in the AD model cells was largely dependent on that in NSCs. These results suggest that the effect of NSCexo on miR-138-5p expression was almost equivalent to that of NSCs.

### MiRNA-138-5p Regulates Tau Expression by Targeting Its 3′UTR

**Fig. 4:**
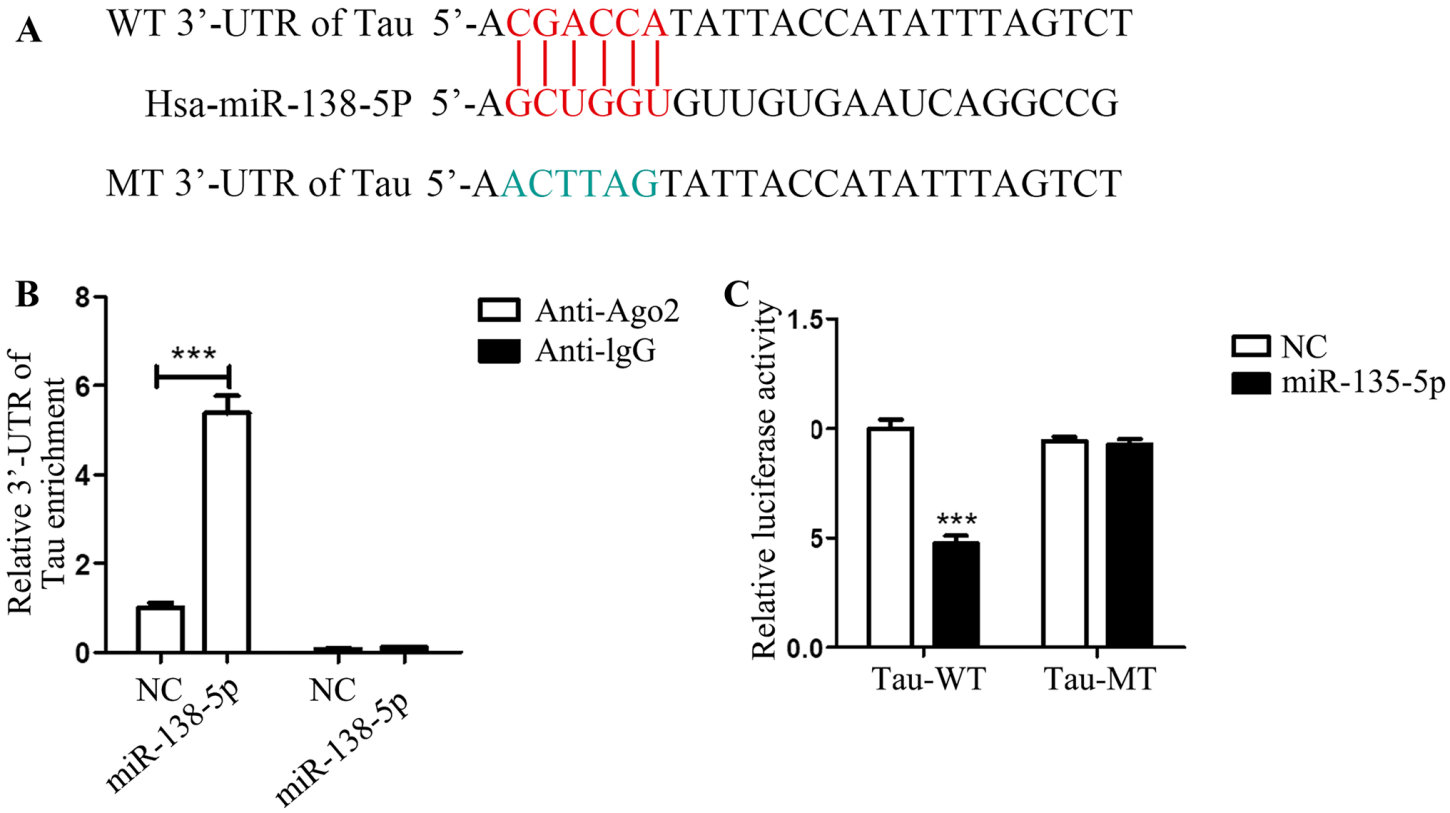
miR-138-5p targeted the 3′UTR of Tau. **A** Representative of the potential complementary sequence between miR-138-5p and the wild-type (WT) 3′UTR of Tau and the mutant (MT) version of 3′UTR of Tau. **B** RIP assay was performed to detect the interaction between miR-138-5p and Tau; Ago2 antibody (Anti-Ago2) was used to pull-down miR-138-5p; Anti-IgG was used as the negative control; NC indicated the scramble miRNA (control of miR-138-5p). **C** Dual-luciferase reporter gene assay was used to detect the interaction between miR-138-5p and the 3′UTR of Tau; Tau-WT indicates the wild-type version of 3′UTR of Tau and Tau-MT indicates the mutant version of 3′UTR of Tau; NC indicated the scramble miRNA which is used as a control of miR-138-5p. ***p < 0.001 vs the indicated control

MiRNA perform its biological function by targeting the 3′UTR of downstream genes; thus, bioinformatics analysis was conducted. We found the 3′UTR of Tau exists a potential complementary sequence to miR-138-5p (Fig. 4A). Based on the binding sites between Tau and miRNA-138-5p, we constructed wild-type and mutant-type Tau vectors. RIP assay revealed a great abundance of miRNA-138-5p binding with anti-Ago2 in 293 T cells but not the negative control IgG (Fig. 4B). Moreover, dual-luciferase reporter gene assay verified that cells co-transfected with miRNA-138-5p mimics and the wild-type Tau 3′UTR (Tau-WT), but not the mutant version of Tau 3′UTR (Tau-MT), showed decreased luciferase activity, suggesting the binding of miRNA-138-5p to the 3′UTR of Tau (Fig. 4C). The above data all demonstrated that Tau was the target gene to bind to miRNA-138-5p directly.

### NSCexo miR-138-5p Inhibits Tau Expression and Reduces Cell Apoptosis of the AD Model Cells

**Fig. 5:**
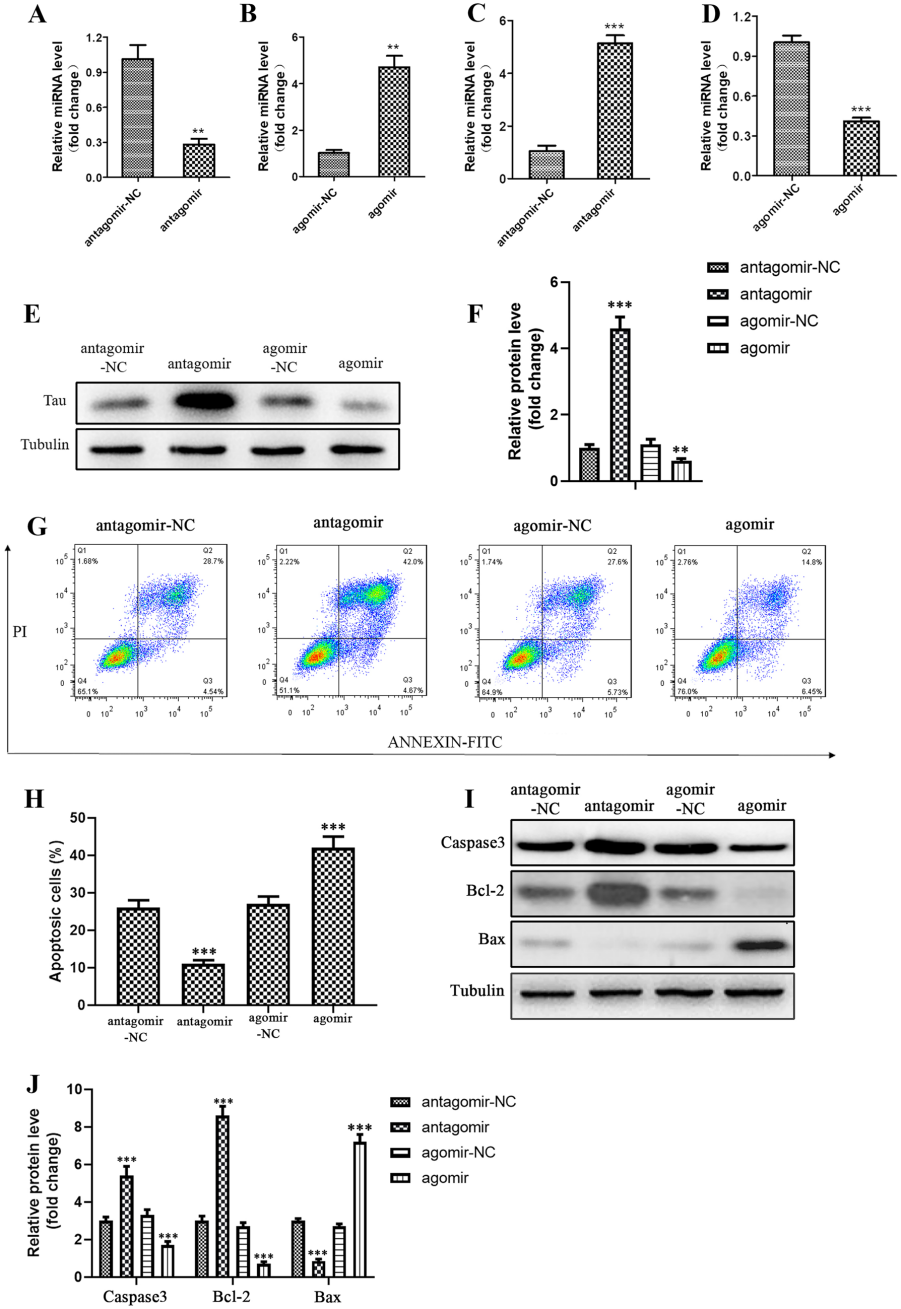
NSCexo miR-138-5p negatively regulates Tau expression and inhibits apoptosis of the AD model cells. **A**, **B** miR-138-5p inhibitor (antagomir) (**A**) or mimics (agomir) (**B**) were administrated to NSC cells for 24 h; the corresponding NSCexo were then co-cultured with the AD model cells for another 24 h, then miR-138-5p level was detected in the AD model cells using QT-qPCR. **C**, **D** QT-qPCR was used to detect Tau expression in the AD model cells that administrated with exosomes that derived from NSCs with miR-138-5p knockdown (**C**) or overexpression (**D**). **E** Western blot was employed to detect Tau accumulation in the indicated cells. Quantification of **E** showed in **F**. **G** Flow cytometry was used to detect the apoptosis of AD model cells that administrated with exosomes that derived from NSCs with miR-138-5p knockdown or overexpression. Quantification of **G** showed in **H**. **I** Western blot was employed to detect the accumulation of apoptotic related markers in AD model cells that supplemented with the corresponding NSCexo. Quantification of **I** showed in **J**. **p < 0.01, ***p < 0.001 vs the indicated control

To further explore the effects of miRNA-138-5p, we inhibited miRNA-138-5p with an antagomir and overexpressed miRNA-138-5p using agomir in NSCs, and assessed the expression of miRNA-138-5p in NSCexo co-cultured AD model cells. The inhibition of miRNA-138-5p in NSCs resulted in a decrease in miRNA-138-5p (Fig. 5A), while overexpression of miRNA-138-5p resulted in an increase in miRNA-138-5p in the AD model cells when cocultured with NSCexo (Fig. 5B). To further investigate this putative relationship between miRNA-138-5p and the Tau in the in vitro AD model, we examined the levels of Tau. The downregulation of Tau in the NSCexo cocultured AD model cells was enhanced when NSCs were pretreated with antagomir, and reversed when pretreated with agomir (Fig. 5C–F). Furthermore, we evaluated the physiological effects of exosomes, from miRNA-138-5p knockdown or overexpression NSCs, on the in vitro AD model. The apoptosis was inhibited with the overexpression of miRNA-138-5p but increased with the downregulation by antagomir (Fig. 5G, H). In addition, the expression levels of the apoptosis-related markers, including caspase3, Bcl-2, and Bax in the in vitro AD cells were detected. As shown in Fig. 5I and J, these makers were significantly downregulated in the miRNA-138-5p-overexpressing NSCexo-treated AD model cells. Overall, these results indicated that NSCexo miRNA-138-5p may inhibit neuronal apoptosis through mediating the accumulation of Tau.

### CA Regulates AD Progression Though NSCexo miR-138-5p

**Fig. 6:**
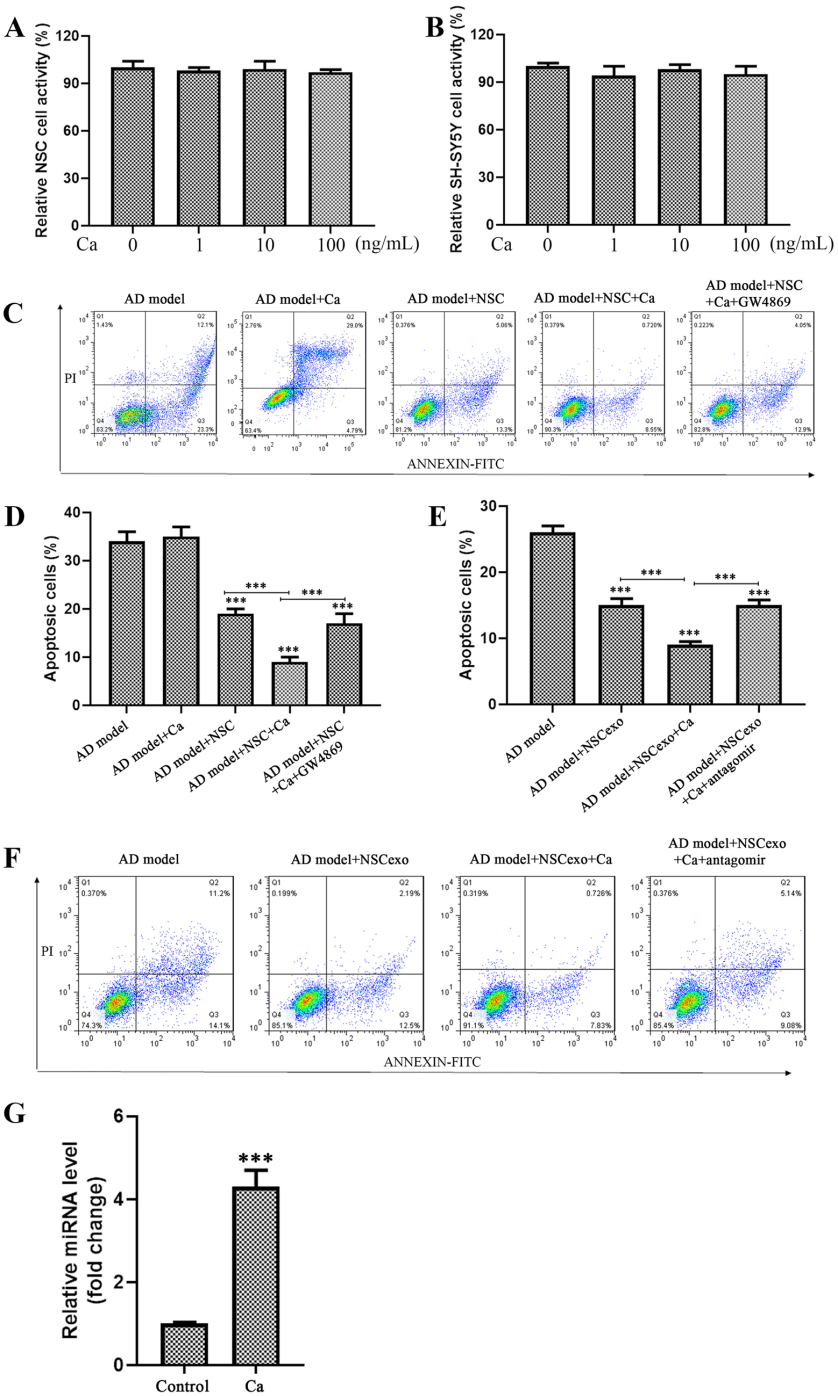
Ca inhibited AD model cell apoptosis by promoting miR-138-5p expression. (**A**, **B**) MTT assay was employed to detect SH-SY5Y (**A**) and NSC (**B**) cell activity after these cells were supplemented with 1, 10, or 100 ng/ml Ca for 48 h. **C** AD model cells were treated with 10 ng/ml Ca alone or together with NSCs, or NSCs + GW4869, 48 h later these cells were subjected to flow cytometry assay to detect cell apoptosis. Quantification of **C** showed in **D**. **F** NSCs were treated with or without 10 ng/ml Ca, or treated with 10 ng/ml Ca plus miR-138-5p knockdown, then the relevant NSCexo was co-cultured with the AD model cells for 24 h, after which flow cytometry assay was performed to detect apoptosis of the indicated AD model cells. Quantification of **F** showed in **E**. **G** NSCs were treated with (Ca) or without (control) 10 ng/ml Ca for 48 h, then the relevant NSCexo was co-cultured with AD model cells for 24 h, after which miR-138-5p level was detected in the AD model cells. ***p < 0.001 vs the indicated control

Our previous report showed that Rehmannia could effectively alleviate the symptoms of AD patients, but the mechanism of action is not clear (Meng et al. 2020). Thus, the underlying mechanism of Rehmannia was explored in the present study. Ca is the main extract of Rehmannia; thus, we assayed whether Ca plays a role in AD progression. We first tested the cytotoxicity of Ca, and found that the cell viability of SH-SY5Y cells and NSCs was not significantly altered with Ca gradient treatment (1, 10, 100 ng/ml) (Fig. 6A, B), proving Ca was not cytotoxic to these cells. Further, we treated the in vitro AD cells with 10 ng/ml Ca and found the cell apoptosis was not significant changed, suggesting that it may not directly act on nerve cells to alleviate the AD progression (Fig. 6C, D). Considering that NSCs play an important role in alleviating the progression of AD, we treated NSCs with Ca (10 ng/ml), and then co-cultured these cells with the in vitro AD model cells. As shown in Fig. 6E and F, the application of Ca significantly enhanced the inhibitory effect on AD model cells apoptosis by NSCs; meantime, GW4869 treatment abolished the effect of Ca. Further, NSCexo were directly added to the AD model cells; as shown in Fig. 6E and F, exosomes from Ca pretreatment NSCs had a stronger inhibitory effect on the apoptosis of the in vitro AD model as compared with PBS pretreated control; these results indicated that Ca inhibits the apoptosis of AD model cells through NSCexo. We further tested the effect of Ca on the expression of miR-138-5p in the in vitro AD model cells. As shown in Fig. 6G, the NSCexo from Ca pretreatment NSCs significantly enhanced the expression of miR-138-5p in the AD model cells, indicating that Ca may mediate AD model cell apoptosis by promoting miR-138-5p level in NSCexo.

### CA Regulates AD Progression by Promoting Neuronal Cell miR-138-5p Expression In Vivo

**Fig. 7:**
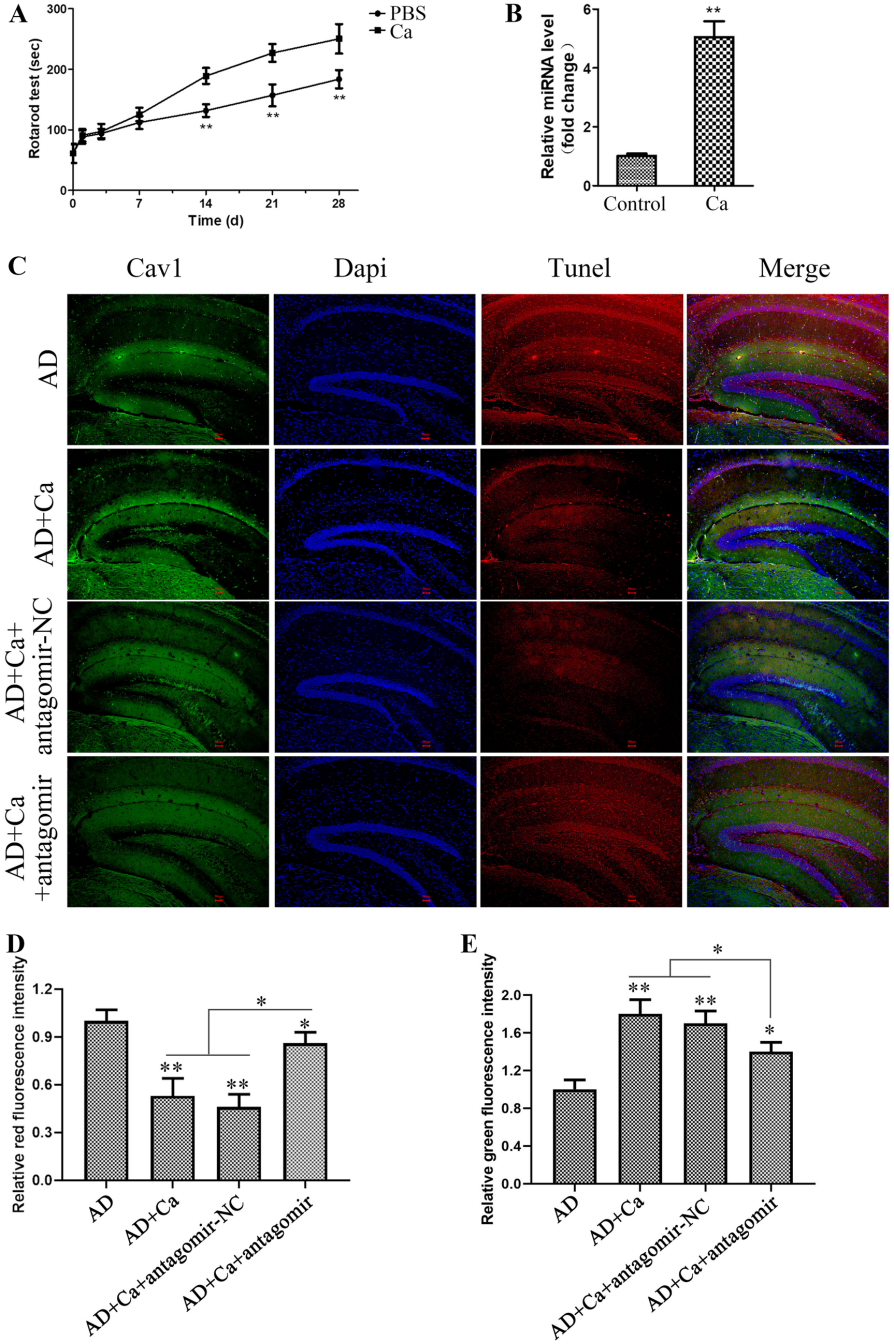
Ca gavage mitigates AD progression in mice. **A** Rotarod test was used to measure the cognitive of the model rats that intragastric administrated (beginning at the 9-week age of the model rats) with PBS (control) or 120 mg/kg Ca daily for 4 weeks. **B** miR-138-5p level detection in the rat hippocampus after Ca administrated for 4 weeks. **C** Co-staining for Cav1, Dapi, and Tunel in the hippocampus after Ca administrated for 4 weeks. **D** Quantification of the red fluorescence intensity of **C**. **E** Quantification of the green fluorescence intensity of **C**. *p < 0.05, ***p < 0.001 vs the indicated control

To further test Ca function in AD progression, we employed an AD mice model. As determined by the Rotarod test (Fig. 7A), Ca administration significantly improved the cognitive performance of the AD mice compared with the control, suggesting Ca is effective in mitigating AD progression. Meantime, miR-138-5p was found significantly upregulated in mouse hippocampus as compared with the PBS-treated control (Fig. 7B). Furthermore, we tested the apoptosis of brain cells and found that Ca administration significantly inhibited the apoptosis of brain nerve cells in hippocampus as determined by the Tunel assay (Fig. 7C, D); however, knockdown of miR-138-5p, but not the negative control antagomir-NC, in hippocampus markedly inhibited the protective function of Ca, which is consistent with the in vitro results. Caveolin-1 (Cav-1) is a membrane/lipid raft (MLR) scaffolding protein necessary for synaptic and neuroplasticity, and play a vital role in maintaining the integrity of neurons. Decreased Cav-1 is related to AD progression and restore Cav-1 level in hippocampus which effectively promotes the cognitive performance of AD mice (Bonds et al. 2019). Thus, we further detected the impact of Ca to the hippocampus Cav-1 level in AD mice, as shown in Fig. 7C and E. Ca administration significantly promoted Cav-1 level but miR-138-5p knockdown mitigated the function of Ca. These results together indicated that Ca mitigated the progress of AD by interfering with the expression of miR-138-5p in nerve cells.

## Discussion

The current Alzheimer’s disease cases worldwide are reported to be around 24 million, and in 2050, the total number of people with dementia is estimated to increase 4 times (Rosenberg et al. 2020). Even though AD is a public health issue, as of now, there is only two classes of drugs approved to treat AD, including inhibitors to cholinesterase enzyme (naturally derived, synthetic and hybrid analogs) and antagonists to N-methyl d-aspartate (NMDA) (Breijyeh and Karaman 2020; Thoe et al. 2021). Several physiological processes in AD destroy Ach-producing cells which reduce cholinergic transmission through the brain. Acetylcholinesterase inhibitors (AChEIs), which are classified as reversible, irreversible, and pseudo-reversible, act by blocking cholinesterase enzymes from breaking down ACh, which results in increasing ACh levels in the synaptic cleft (Liss et al. 2021; Srivastava et al. 2021). On the other hand, overactivation of NMDAR leads to increasing levels of influxed Ca^2+^, which promotes cell death and synaptic dysfunction. Despite the therapeutic effect of these two classes, they are effective only in treating the symptoms of AD, but do not cure or prevent the disease. Unfortunately, only a few clinical trials on AD have been launched in the last decade and their outcome was unsatisfied (Babiloni et al. 2021; Hung and Fu 2017). Compared with the monomer medicine, Traditional Chinese medicine compounds have shown unique advantages because of their function on multiple targets and multiple pathways (Klimova and Kuca 2017; Shi et al. 2017). Our previously study have revealed the protecting role of Rehmannia against AD progression (Meng et al. 2020); however, the complex components and unclear mechanisms have apparently limited its application; thus, identifying the active ingredient and exploring the underlying mechanisms of Rehmannia function is extremely important.

Ca is a water-soluble active ingredient isolated from the root of Rehmannia, and exhibits multiple pharmacological activities such as anti-inflammation (Zheng et al. 2017), antioxidation (Zheng et al. 2017), and anti-apoptosis (Liu et al. 2017), and shown neuroprotective properties against hypoxic/ischemic injury (Jiang et al. 2015); thus, it is interesting to explore whether Ca plays a role in AD progression. As expected, Ca administration could effectively inhibit the in vitro AD cell apoptosis in a NSCexo-dependent manner, and more importantly Ca administration significantly inhibited cell apoptosis in hippocampus and promoted the cognitive performance of AD mice. Using the in vitro AD model, we found directly administrated Ca to the in vitro AD cells could not inhibit cell apoptosis; however, after pretreated with Ca, the protective efficiency of NSCs or NSCexo on AD model cells was significantly enhanced, but this tendency was abolished by the exosome inhibitor GW4869, suggesting Ca functions though NSCexo.

Exosomes released from activated or apoptotic cells contain specific proteins (signaling molecules, receptors, integrins, cytokines), bioactive lipids, and nucleic acids (mRNA, miRNA, small noncoding RNAs, DNA) from their progenitor cells (Samanta et al. 2018; Théry et al. 2002). MiRNAs, a class of small noncoding transcripts, are known to play important roles in development, metabolism, and neural plasticity. Studies have shown that dysregulation of miRNA may play a complex role in AD (Delay et al. 2012). Thus, to further explore the function underlying NSCexo, we focused on the miRNAs that relevant to nerve cell function. miR-138-5p was previously reported function in neural stem cell proliferation and differentiation (Wang et al. 2017), regulates Schwann cell migration and proliferation (Liu et al. 2022), participates in inflammatory response during cerebral ischemia–reperfusion injury (Li et al. 2020), and favors neural cell survival in the injured spinal cord by targeting pro-apoptotic factors (Maza et al. 2022). Thus, it is not a surprise that miR-138-5p regulates AD progression. Indeed, miR-138-5p play a central role in Ca and NSCexo function, because Ca administration promoted NSCexo miR-138-5p level, and knockdown miR-138-5p in NSCs effectively mitigated Ca function in the in vitro model. More importantly, knocking down miR-138-5p in the hippocampus of AD mouse significantly inhibits the anti-apoptotic function of Ca.

We further explored the downstream factor of miR-138-5p, and found miR-138-5p could directly interact with the 3′UTR of Tau and negatively regulates its expression. As all known, Tau overaccumulation in neurons is one of the main reasons for AD progression (Dujardin et al. 2014; Mershin et al. 2004); thus, it is possible that Ca protects against AD progression by inhibiting miR-138-5p-mediated Tau accumulation. Cav-1, a MLR scaffolding protein, is another factor that plays a vital role in AD progression (Bonds et al. 2019). It regulates the integrity of neurons and plays a fundamental role for synaptic and neuroplasticity. Recent researches have demonstrated that decreased Cav-1 is significantly related to AD progression and promoted Cav-1 level by transgenic manner in hippocampus effectively ameliorated the cognitive performance of AD mice (Wang et al. 2021). In the present study, we found that the level of Cav-1 in the hippocampus of AD mice was upregulated after Ca treatment, whereas significantly downregulated upon miR-138-5p knockdown, suggesting Ca may also mediate AD progression though miR-138-5p/Cav-1 axis. However, after searched the gene sequence of Cav-1, no potential sequence to pair with miR-138-5p exists, suggesting miR-138-5p may not be a direct regulator of Cav-1.

Overall, our data demonstrate that the delivery of NSCexo reduces the apoptosis response in AD and revealed that NSCexo miR-138-5p acted as a protective factor for AD by targeting Tau. Furthermore, we demonstrated that Ca, the extract of traditional Chinese medicine Rehmannia, could perform its protective role in AD progression both in vitro and in vivo by mediating miR-138-5p expression.

## Supplementary Information

Below is the link to the electronic supplementary material.

Supplementary file1 (JPG 722 KB)

## Data Availability

The datasets generated during and/or analyzed during the current study are available from the corresponding author on reasonable request.
