# Supplementary figures and images for: Catalpol Mitigates Alzheimer's Disease Progression by Promoting the Expression of Neural Stem Cell Exosomes Released miR-138-5p
Source: Neurotox Res. 2023 Jan 3;41(1):41–56. doi: 10.1007/s12640-022-00626-z (PMC9944361; doi:10.1007/s12640-022-00626-z)

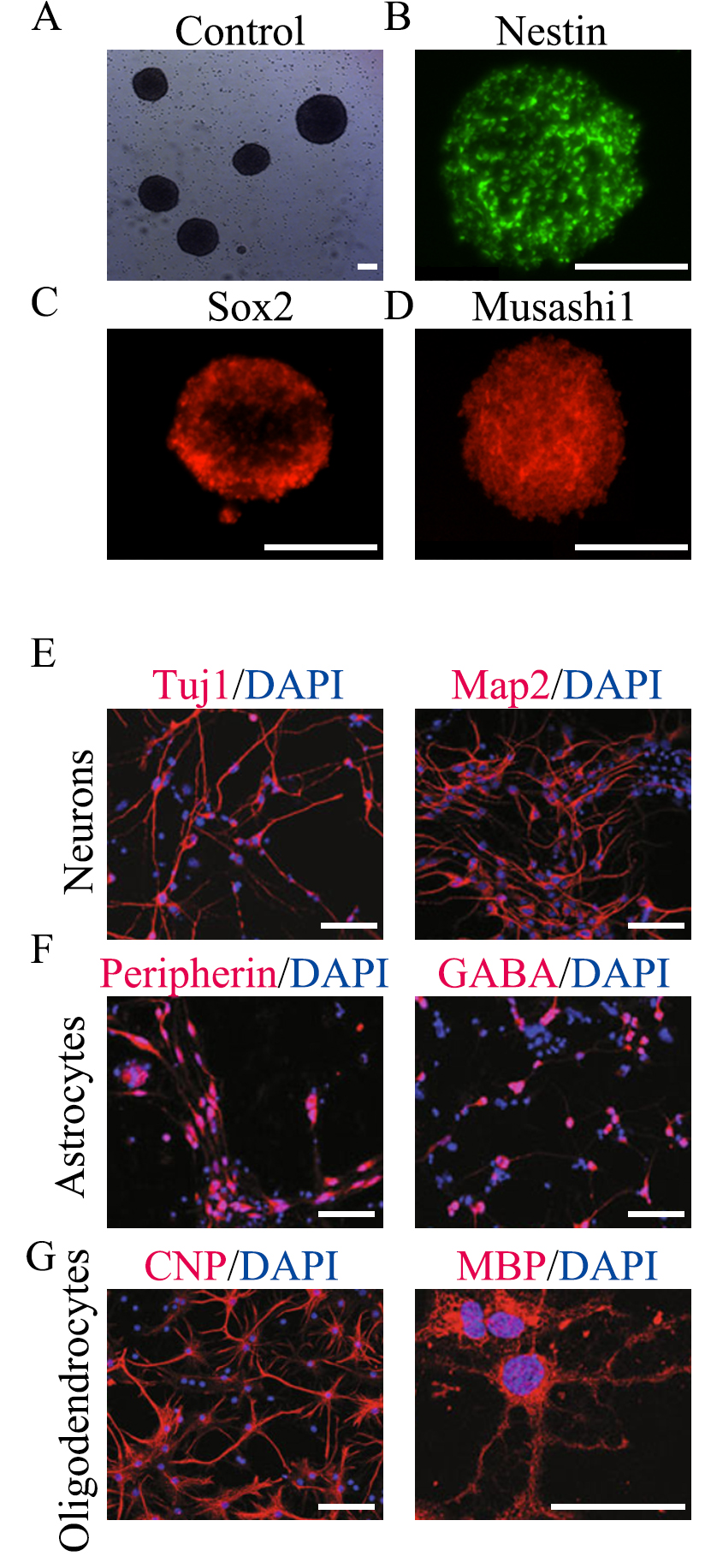

Supplement: Supplementary file 1 — Supplementary file1 (JPG 722 KB) [file 12640_2022_626_MOESM1_ESM.jpg]
